# Hypermethylation of OPRM1: Deregulation of the Endogenous Opioid Pathway in Myalgic Encephalomyelitis/Chronic Fatigue Syndrome and Fibromyalgia

**DOI:** 10.3390/ijms27020826

**Published:** 2026-01-14

**Authors:** Arne Wyns, Jolien Hendrix, Jente Van Campenhout, Yanthe Buntinx, Huan-Yu Xiong, Elke De Bruyne, Lode Godderis, Jo Nijs, David Rice, Daniel Chiang, Andrea Polli

**Affiliations:** 1 Pain in Motion (PiM) International Research Group, Department of Physiotherapy, Human Physiology and Anatomy, Faculty of Rehabilitation Sciences & Physiotherapy, Vrije Universiteit Brussel, Laarbeeklaan 103, 1090 Jette, Belgium; 2 Department of Public Health and Primary Care, Centre for Environment & Health, KU Leuven, Kapucijnenvoer 35, 3000 Leuven, Belgium; 3 Flanders Research Foundation—FWO, 1000 Brussels, Belgium; 4 Translational Oncology Research Center (TORC), Team Hematology and Immunology (HEIM), Vrije Universiteit Brussel, 1090 Jette, Belgium; 5 External Service for Prevention and Protection at Work, IDEWE, 3001 Heverlee, Belgium; 6 Department of Physical Medicine and Physiotherapy, University Hospital Brussels, 1000 Jette, Belgium; 7 Institute of Neuroscience and Physiology, University of Gothenburg, 405 30 Gothenburg, Sweden; 8 Health and Rehabilitation Research Institute, Auckland University of Technology, Auckland 1023, New Zealand; 9 Department of Anaesthesiology, Perioperative & Pain Medicine, Health New Zealand, Te Whatu Ora Waitemata, Auckland 0620, New Zealand; 10 Department of Pharmacology and Clinical Pharmacology, Faculty of Medicine and Health Science, University of Auckland, Auckland 1023, New Zealand; 11 Department of Anaesthesiology, Faculty of Medicine and Health Science, University of Auckland, Auckland 1023, New Zealand

**Keywords:** myalgic encephalomyelitis/chronic fatigue syndrome, fibromyalgia, opioids, OPRM1, descending modulation, epigenetics, bisulfite conversion

## Abstract

Myalgic encephalomyelitis/chronic fatigue syndrome (ME/CFS) and fibromyalgia (FM) are debilitating disorders with overlapping symptoms such as chronic pain and fatigue. Dysregulation of the endogenous opioid system, particularly µ-opioid receptor function, may contribute to their pathophysiology. This study examined whether epigenetic modifications, specifically µ-opioid receptor 1 gene (*OPRM1*) promoter methylation, play a role in this dysfunction. Using a repeated-measures design, 28 ME/CFS/FM patients and 26 matched healthy controls visited the hospital twice within four days. Assessments included blood sampling for epigenetic analysis, a clinical questionnaire battery, and quantitative sensory testing (QST). Global DNA (hydroxy)methylation was quantified via liquid chromatography–tandem mass spectrometry, and targeted pyrosequencing was performed on promoter regions of *OPRM1*, *COMT*, and *BDNF*. ME/CFS/FM patients reported significantly worse symptom outcomes. No differences in global (hydroxy)methylation were found. Patients showed significantly higher *OPRM1* promoter methylation, which remained after adjusting for symptom severity and QST findings. Across timepoints, *OPRM1* methylation consistently correlated with *BDNF* Promoter I and Exon III methylation. This is, to the best of our knowledge, the first study examining *OPRM1* methylation in ME/CFS/FM. Increased *OPRM1* methylation in patients, independent of symptoms or pain sensitivity measures, supports the hypothesis of dysregulated opioidergic signaling in these conditions.

## 1. Introduction

Myalgic encephalomyelitis/chronic fatigue syndrome (ME/CFS) and fibromyalgia (FM) are two separate conditions characterized by disabling fatigue, post-exertional malaise (PEM), and chronic widespread pain, respectively [1]. However, the two conditions have a similar clinical presentation with overlapping symptoms such as cognitive impairment, sleep alteration, and mood disturbance [2,3]. Moreover, approximately 70% of ME/CFS patients also meet the diagnostic criteria for FM, and this comorbidity best discriminates ME/CFS patients with higher symptom burden and disability as compared to patients with ME/CFS alone [4]. Both conditions predominantly affect women, with ME/CFS affecting approximately 1% of the global population and FM affecting up to 5% [5,6]. Without effective treatment options, these disorders are associated with a high socioeconomic burden, and therefore, unravelling the underlying mechanisms of ME/CFS and FM is urgently needed.

Despite an increasing amount of research, both disorders remain poorly understood in terms of pathophysiological mechanisms and cannot be diagnosed using objective measures [7]. Recent research has highlighted the potential role of the autonomic nervous system [8], neuro-immunological dysregulation [9,10], oxidative stress alterations [11,12,13], and dysregulation of the descending nociceptive modulatory pathways in ME/CFS and FM aetiology [14]. Furthermore, it seems that a dysregulated endogenous opioid system is of major relevance, given the important role of the endogenous opioidergic pathways in descending nociceptive modulation [15] and the observation that patients with FM have paradoxically elevated levels of endogenous opioids in the cerebrospinal fluid [16].

Endogenous and exogenous opioids exert their antinociceptive function by binding to the µ-opioid receptor (MOR) in brain regions that give rise to descending nociceptive modulatory pathways [17]. An early study found that patients with FM exhibit decreased MOR availability in the nucleus accumbens and the amygdala [18]. The same research group found a strong association between decreased MOR binding potential and reduced pain-evoked brain activity in these patients using combined positron emission tomography (PET) and functional magnetic resonance imaging (fMRI) [19].

Similar effects can be observed in opioid use disorders, where chronic use or abuse of opioids leads to desensitization and downregulation of opioid receptors [20]. Patients with a history of heroin use and currently on methadone maintenance treatment, as well as prior opioid users, showed increased methylation of the CpG island spanning the promoter region of the *OPRM1* gene (the gene encoding MOR) in peripheral leukocytes [21,22]. Hypermethylation of this promoter region has been shown to transcriptionally silence *OPRM1* expression [23]. Moreover, a recent study by Sandoval-Sierra et al. provided insights on the development of *OPRM1* promoter hypermethylation in opioid-naïve participants, who were prescribed opioids after dental surgery. They found 9 out of 10 selected CpG sites in the promoter region to be hypermethylated in participants receiving high doses of opioids compared to lower doses, 40 days after surgery [24]. Another study found that methylation around the *OPRM1* transcription start site could predict the development of both acute and chronic post-surgical pain after spine fusion surgery [25]. For all CpG sites analysed, higher methylation was associated with higher odds of developing acute or chronic post-surgical pain. These studies provide compelling evidence that exposure to endo- and exogenous opioids, short-term and prolonged, can influence the epigenetic landscape of and around the *OPRM1* promoter and that this landscape could predict the development of chronic pain [25,26].

Previous research from our group explored methylation profiles of two other prominent genes that regulate nociceptive modulatory pathways, Catechol-O-Methyltransferase (*COMT*) and Brain-Derived Neurotrophic Factor (*BDNF*), respectively. Results showed higher methylation on the promoter of the membrane-bound version of *COMT* (*MB-COMT*) and lower methylation on the *BDNF* coding region (Exon 9) in patients with ME/CFS/FM compared to healthy controls [27,28]. *BDNF* promoter hypomethylation was accompanied by higher serum BDNF concentrations in these patients. On the one hand, COMT may be, although less pronounced compared to *OPRM1*, involved in descending modulation pathways through its effects in the autonomic nervous system [27]. BDNF, on the other hand, has been shown to regulate the induction and maintenance of central sensitization [28,29].

Despite its critical role in nociceptive regulation, the endogenous opioid system remains surprisingly underexplored in ME/CFS/FM. We hypothesise that the opioid system of patients with ME/CFS and FM is dysregulated and that *OPRM1* epigenetic regulation, at least partially, drives this dysregulation and may account for poorer clinical outcomes. The present work aims to investigate the methylation profile of the *OPRM1* promoter in ME/CFS and FM and explore associations with the major symptom cluster and measures of pain sensitivity and descending pain modulation.

## 2. Results

### 2.1. Clinical Characteristics

A total of 54 women, 28 patients with ME/CFS/FM and 26 healthy controls, participated in the study. Both body mass index (BMI) and physical activity were comparable between groups (*p* > 0.05). Although participants were age- and BMI-matched, mean age differed between groups, with the CFS/FM group being slightly younger than the control group (*p* = 0.023). This is a common and acceptable effect of the frequency-based matching approach, which does not guarantee equal means. Nevertheless, inclusion of both age and BMI as covariates in downstream analysis is warranted. All outcomes of the clinical questionnaire differed significantly between groups, with CFS/FM patients reporting higher scores on the Short Form-36 (SF-36), Widespread Pain Index (WPI), Central Sensitization Inventory (CSI), Beck Anxiety Inventory (BAI), and Pain Catastrophizing Scale (PCS) (*p* < 0.001). Detailed characteristics for group differences are summarized in Table 1. Between-group differences in clinical objective pain thresholds and temporal summation have been previously described [27,28].

### 2.2. Global Methylation and Hydroxymethylation Remained Unchanged

To investigate the impact of CFS/FM on the global methylome, we analyzed the global levels of 5-Methylcytosine (5mC) and 5-hydroxymethylcytosine (5hmC). Linear mixed-effect models were fitted with 5mc and 5hmC as dependent variable. The models were corrected for both age and BMI. No between-group differences in 5mC and 5hmC were observed (see Table 2).

### 2.3. Patients with CFS/FM Present Higher Methylation of the OPRM1 Promoter

Separate mixed models were fitted for the three CpG site primers. Additionally, the mean methylation was calculated as a more representative measure of the region. Two of the three CpG sites showed significantly higher methylation in the CFS/FM group compared to the healthy controls (Table 2), which translated to a significant difference in mean *OPRM1* methylation (univariate test for group: F = 5.136; mean difference = 0.524 [95% C.I. 0.065–0.983]; *p* = 0.026). In the latter model, age was significantly associated (weak positive association) with *OPRM1* methylation, suggesting that *OPRM1* methylation increases with age (F = 4.040; regression coefficient = 0.023 [95% C.I. 0.00–0.045]; *p* = 0.047).

### 2.4. OPRM1 Methylation Is Not Associated with the Composite Symptom Factor

We used the extracted principal component analysis (PCA) component in a linear mixed model to investigate whether *OPRM1* methylation was associated with the composite symptom factor. The between-group difference was stable, with the patient group showing higher *OPRM1* methylation compared to healthy controls (univariate test for group: F = 5.205; mean difference = 0.985 [95% C.I. 0.129–1.842]; *p* = 0.025). The composite symptom factor was not associated with *OPRM1* methylation (fixed-effect estimate = −0.269 [95% C.I. −0.693–0.154]; std. error = 0.213; *p* = 0.210).

### 2.5. OPRM1 Methylation Was Not Associated with Measures of Pain Sensitivity or Descending Pain Modulation

The thermal pain thresholds were strongly correlated, but the analysis revealed an evident ceiling effect. Therefore, predicted values were calculated by fitting the three cold and heat pain thresholds in two separate regression models. These predicted values represent cold and heat pain sensitivity, respectively [27]. Between-group comparisons of thermal and pressure pain thresholds revealed significant differences, indicating that patients had higher sensitivity to cold (univariate test for group: F = 14.178; mean difference = 3.719 [95% C.I. 1.759–5.678]; *p* < 0.001), heat (univariate test for group: F = 11.794; mean difference = −2.011 [95% C.I. 0.849–3.172]; *p* < 0.001), and pressure stimuli (univariate test for group: F = 21.691; mean difference = −1.155 [95% C.I. 0.663–1.647]; *p* < 0.001) (see Table 3). When we investigated whether *OPRM1* methylation was associated with pain thresholds, no observable effect was found on pain thresholds (see Table 4). A linear mixed model with Conditioned Pain Modulation (CPM), a paradigm to measure descending pain modulation, as dependent variable, time as repeated measure, and age and BMI as covariates, revealed a significant between-group difference of CPM to cold stimuli, suggesting the patient group had a larger CPM response compared to the healthy controls (univariate test for group: F = 6.667; mean difference = 1.070 [95% C.I. 0.247–1.893]; *p* = 0.011). No differences were observed for CPM to heat stimuli. When a linear mixed model was fitted with *OPRM1* methylation as dependent factor and both CPM outcomes as covariates, the between-group difference in methylation disappeared (see Table 4).

### 2.6. OPRM1 Methylation Is Highly Correlated with Methylation of the First Promoter of BDNF

Exploratory correlation analysis was performed to investigate whether DNA methylation of *OPRM1*, *BDNF*, and *COMT* were associated (see Table 5). We observed a significant strong positive correlation of *OPRM1* and *BDNF* promoter 1 (3/4 amplicons) and exon 3 (2/2 amplicons) methylation at both timepoints (see Table 5). Interestingly, we observed significant weak-to-moderate negative correlations between *OPRM1* and two of the three amplicons from the *S-COMT* promoter and with Exon IV of the *COMT* gene. This observation was only found at the first timepoint.

## 3. Discussion

To the best of our knowledge, this is the first study investigating *OPRM1* methylation in ME/CFS and FM. We employed a repeated-measure design to internally control our measurements and account for intra-subject variability. Our main objective was to investigate whether *OPRM1* was differentially methylated in ME/CFS/FM patients and whether these changes were associated with clinical and neurophysiological measures.

ME/CFS/FM patients reported significantly worse symptom outcomes as compared to healthy controls. Additionally, pain threshold measurements showed that these patients had a significantly higher pain sensitivity compared to their matched counterparts. However, patients showed significantly greater CPM to cold stimuli, which seems to be not in line with the existing literature [30]. These findings are counterintuitive and led us to question the validity and objectivity of CPM paradigms to assess endogenous nociceptive modulation. A recent review by Carneiro et al. highlights the heterogeneity in Quantitative Sensory Testing (QST) methodology, which negatively influences the interpretation and comparability of studies but also affects the reproducibility of future studies [31]. In addition, other confounding factors such as reporting bias, participant awareness and attention, and test–retest reliability in this population could potentially explain these anomalous results. While we included substantive assessor training in performing standardized procedures to limit assessor, reporting, and scoring bias, we could not fully mitigate the effect of patient awareness and attention, apart from instructing the participant to concentrate during testing. The test–retest variability of our QST results was previously reported and revealed excellent test–retest reproducibility using Interclass Correlation Coefficient (ICC) analysis [32]. Although a substantial effort was made to limit the effect of possible confounders, the eventual effect cannot be underestimated as this may lead to a Type I error. No difference in CPM to heat stimuli was detected.

We found significantly higher methylation on the *OPRM1* promoter in CFS/FM patients compared to healthy controls, which is in line with our hypothesis. Hypermethylation of the *OPRM1* promoter has previously been shown to transcriptionally silence MOR expression [23]. *OPRM1* hypermethylation led to the recruitment of methyl-CpG-binding proteins (MeCP2), which in turn recruited repressors (HDAC1 and mSin3A), ultimately leading to histone deacetylation and nucleosome reformation to a compacted inactive promoter [23,33]. A more recent study found that, in acute and neuropathic pain, *OPRM1* expression was effectively inhibited by the methyl-CpG-binding domain 1 (MBD1) protein-mediated recruitment of the de novo DNA methyltransferase 3a (DNMT3a) enzyme [34]. Epigenetic writers like DNMTs have gained attention for their potential role in modulating nociceptive processes [35]. Another study in heroin addicts found, however, that the *OPRM1* promoter was also hypermethylated, with increased density around the Sp1 transcription factor binding site, directly inhibiting the binding of the transcriptional machinery [21]. These studies suggest multiple ways in which *OPRM1* promoter hypermethylation may potentially interrupt expression and, by extension, downstream opioidergic pathways. While informative, these mechanisms of hypermethylation-mediated transcriptional silencing remain speculative, and further research should include functional assays to validate their involvement.

When analysing associations with clinical and neurophysiological variables, we found that the between-group difference in methylation was robust when added to the model. However, no associations between *OPRM1* methylation and the composite symptom factor and QST measurements were measured. Similarly, CPM measurements were not associated, and the between-group methylation difference disappeared. Apart from the central role of *OPRM1* in descending pain modulation, methylation alone may be insufficient to significantly affect the endo-opioidergic functions. It is likely that other pathways and mechanisms play significant roles in this complex phenomenon, which may explain the apparent lack of associations found here. One novel hypothesis focuses on other epigenetic mechanisms, such as histone markers and microRNAs [36,37]. Another focuses on the monoamine (dopamine, serotonin, and adrenergic) signalling pathways [38], in addition to the opioidergic system, which have been shown to affect CPM measures in paediatric patients with chronic low back pain [39].

Interestingly, we found a significant strong-to-moderately positive correlation between *OPRM1* promoter methylation and *BDNF* promoter I and exon III methylation that was stable over both timepoints. The role of BDNF in chronic pain has been widely studied and a recent study reviewed the multiplicity, some counteracting, effects of this polyvalent protein [32]. Among its pain-related effects, BDNF is particularly implicated in the induction and maintenance of central sensitization, a process in which neurological responses to nociceptive stimuli are amplified [29]. Previously, differences in *BDNF* methylation have been described as part of this study [28]. A correlation between *OPRM1* and *BDNF* on the epigenetic level has not yet been described. In contrast, a possible link at the genetic level has been suggested by a recent study in patients with chronic knee osteoarthritis, which shows that carriers of the clinical polymorphisms in both the *OPRM1* (A118G) and *BDNF* (G196A) genes were less responsive to rehabilitation-induced analgesic response [40]. Variation in genetic polymorphisms can influence DNA methylation around the polymorphism itself and thus contribute to gene expression [41]. Both *OPRM1* and *BDNF* have several clinically relevant polymorphisms that have been studied [42]. Unfortunately, we were not in a position to investigate *OPRM1* polymorphisms during this study.

Lastly, Global DNA (hydroxy)methylation remained unchanged between groups, suggesting sensitive and highly contextual methylation changes at specific CpG sites (i.e., local changes). This is consistent with a study by Trivedi et al., reporting similar global methylation levels between patients with ME/CFS and healthy controls, yet microarray analysis revealed highly contextual differentially methylated CpG sites in regulatory and coding regions [43]. This contextual methylation hypothesis is in line with our findings reporting significantly higher methylation on the *OPRM1* promoter.

As confounding factors, age and BMI have been shown to have a significant effect on epigenetic outcomes, as well as on symptom-related outcomes [44]. Age consistently had a significant effect in our fitted models, suggesting that age could, at least partially, drive this difference. Early evidence suggested that global DNA methylation decreased from adulthood, with the idea that this drives the genome to a more pathologically prone state, also called “epigenetic drift” [45]. In contrast, recent evidence suggests that the genome evolves with more site-specific changes that have highly contextual effects [46]. To date, no evidence on age-related effects on *OPRM1* methylation exists. Nevertheless, these findings underline the importance of accounting for age-related effects when investigating DNA methylation.

We acknowledge that our study is not without limitations. The sample size was primarily calculated to measure differences in *OPRM1* methylation, not global (hydroxy)methylation. These results should, therefore, be interpreted with the necessary caution. Additionally, we analysed three CpG sites in the *OPRM1* promoter region, which contains a CpG island consisting of approximately 50 CpG sites. Although we selected these three CpGs based on the literature [22,25], this “snapshot” approach is a limitation of the current methodology, as this may miss biologically relevant methylation changes elsewhere in the regulatory region. Future research should focus on mapping the epigenome on a larger scale, sequencing longer amplicons that span whole CpG islands or even entire genes. This goes in hand with the changing gold standard of methyl conversion, which to date has been bisulfite conversion. However, this method has been shown to elicit considerable amounts of DNA damage, resulting in low-quality DNA, difficulties in library preparation, and analysis of shorter amplicon sequences [47]. Only recently have more DNA-sparing methods, such as Enzymatic Methyl-sequencing (EM-seq) and TET-assisted pyridine borane sequencing (TAPSß), become available [48]. These methods rely on more gentle enzymatic processes as compared to harsh chemical reactions, resulting in high-quality DNA and long-read amplicons for sequencing [49]. Another limitation is that we used epigenetic markers in peripheral blood cells as a measure for the epigenetic state in the central nervous system. While early interpretations on the epigenetic signature led us to believe that these markers are and remain solely tissue-specific, more recent evidence suggests that the epigenetic signatures of the central nervous system and those of peripheral cells show more resemblance than expected [50,51]. More specifically, peripheral immune cells not only share this general resemblance but also have been proven to express the whole array of opioid receptors [52,53]. Together with the proven effect of opioids on the immune system, this suggests that peripheral immune cells at least partially represent the epigenetic signature of the central nervous system [54,55]. Nevertheless, we recognize that this remains a general limitation of epigenetic research in conditions where target organs are inaccessible and that these reports should be interpreted with caution. Lastly, no transcriptional (mRNA) or translational (protein) analyses were performed. The absence of any functional readouts severely limits the biological interpretation of our results. Generally, increased methylation in the gene promotor region results in transcriptional silencing. Although we assume hypermethylation as a proxy for transcriptional silencing, this cannot be proven without any functional assays. The above-mentioned mechanisms of hypermethylation-mediated transcriptional silencing, while informative, remain speculative in nature. Investigations on the mRNA and protein level will provide greater insights in the biological mechanisms involved. Evidently, it is the combination of the three levels that will provide the most compelling evidence and should therefore be included in further research efforts.

To progress in unravelling these mechanisms, we must emphasize the heterogeneous socioeconomic, psychological, and biological nature of the conditions, the latter of which is likely marked by overlapping, compensatory, and sometimes opposing mechanisms, adding an additional layer of heterogeneity. Because of this heterogeneity, it is of the utmost importance that future studies are well designed, incorporating standardised state-of-the-art methodology and a well-characterized population. Further fundamental research on the intricate ME/CFS/FM condition is warranted and could potentially reveal novel targetable mechanisms.

In conclusion, patients with ME/CFS and FM demonstrated significantly increased methylation in the OPRM1 promoter region compared to healthy controls. This strengthens our hypothesis that the opioidergic system may be dysregulated in these patients. However, further research must reveal how our findings tie into the complex pathophysiological mechanisms underlying ME/CFS/FM.

## 4. Materials and Methods

### 4.1. Study Design

We conducted a repeated-measures study with three days in between the two assessments, designed to serve as internal validation and to control for within-subject variability of biological and neurophysiological measurements. This study was approved by the Medical Ethical Committee of the University Hospital Brussels (ref. 2016/134, approved on 8 July 2016). Data collection was performed at the Department of Internal Medicine and Endocrinology of the University Hospital Brussels from August 2015 to March 2017.

All participants presented to the hospital for study assessment on two separate mornings (between 9 and 11 a.m.), four days apart. Informed consent was obtained before the first assessment was initiated. During the initial assessment, a general intake interview was conducted to collect data concerning general health, comorbidities, and drug intake. All participants were asked to complete questionnaires to assess physical activity and symptom characteristics (refer to Section 4.4 for an overview of the questionnaires). Afterwards, blood samples were drawn, processed within the hour, and stored at −80 °C for further analysis. Finally, neurophysiological assessments were performed as described below. Four days later, all participants underwent the second assessment, identical to the first, that is, questionnaires, blood sampling, and neurophysiological measurements, in that standardized order. Assessments prone to assessor bias, such as the neurophysiological measurements, were performed blinded to group allocation. Even though the assessor was blinded to group allocation, these measurements are prone to both recording and scoring bias. To minimize the bias effect, the assessor was trained and instructed to perform these measurements in a standardized manner and without participant interference. Scoring was performed by the participants, and the assessor remained impartial to the scoring. Although participants were instructed to concentrate during the procedure, factors such as participant awareness and attention could not be fully mitigated. Together, the blinding and standardized execution allowed us to minimize potential bias effects.

Our previous work exploring different (epi)genetic mechanisms on the same samples has been recently published elsewhere [27,28]. The present manuscript explores novel biological mechanisms for ME/CFS and FM (global DNA methylation and hydroxyl-methylation, as well as *OPRM1* DNA methylation) and how these relate to condition symptomology, pain sensitivity, and descending pain modulation. As DNA methylation in other genes (namely, *BDNF* and *COMT*) have been previously explored in this same cohort of patients, we will test associations between global DNA methylation and hydroxyl-methylation and DNA methylation in the *OPRM1*, *BDNF*, and *COMT* genes.

### 4.2. Participants

Patients with a clear diagnosis of ME/CFS, according to the Centre for Disease Control and Prevention Criteria, and FM, according to the American College of Rheumatology Criteria (ACR-2011), were enrolled in this study [31,32]. Healthy controls were recruited among friends or acquaintances of patients or of people involved in the study and were enrolled within the same time period and from the same geographical region as the patients. Only female participants were included in this study due to the higher prevalence of ME/CFS and FM in the female sex and important sex-related differences in pain and immune regulation [33,34]. Patients were instructed to not start new therapies 6 weeks before the first test moment and to not use nicotine, alcohol, and caffeine 24 h before the test moment. Additionally, all participants were screened for comorbidities, and current medication plans were noted. Information on the most prominent medication classes taken by each group can be found in Supplementary Table S1. Potential participants that presented with other neurological, systemic, psychiatric, cardiovascular, or oncological comorbidities were excluded. Patients suffering from ME/CFS and FM are generally less physically active, and because physical activity has been shown to influence the endogenous opioid system, the immune system, and the autonomic nervous system, we selected sedentary participants only. We defined this lifestyle as people with a seated job or hobby and not more than 2 h of moderate activity per week [35]. Additionally, we asked all participants to refrain from engaging in physical exercise one day before and during the assessment period. Patients and healthy controls were matched for age and BMI following the frequency matching approach, checking the variable of interest (age and BMI) frequency for every 5 participants enrolled.

### 4.3. Sample Size Calculation

No previous study has investigated *OPRM1* DNA methylation in ME/CFS and FM. One relevant study, which compared *OPRM1* methylation between chronic pain patient with prolonged (1 year) opioid use (n = 62) and chronic pain patients with no previous history of opioids (n = 70), was used for further sample size calculation [22]. *OPRM1* methylation was significantly higher (*p* = 0.01) in the chronic pain group with opioids (15.81% ± 9.76) as compared to the chronic pain without opioids (12.23% ± 5.10). This is in line with previously published studies; as summarized by Jones et al., the majority of studies show that a mean difference of 1–2% in DNA methylation sufficiently induces downstream gene expression effects [56]. The extracted results equate to a medium effect size (Cohen’s *f*) of 0.230. Using G*Power 3.1 (latest ver. 3.1.9.7; Heinrich-Heine-Universität Düsseldorf, Düsseldorf, Germany), we calculated that to detect an effect size *f* = 0.230 at a significance level α = 0.05 and a power = 80%, employing our repeated-measures design (2 measurements) with 2 groups, and assuming a correlation among repeated measures of 0.3, the total sample size needed was 54 participants. This is in line with results from our previous reports, which showed that 54 participants are sufficient to detect significant between-group DNA methylation differences with a medium effect size in this population [27,28].

### 4.4. Clinical and Neurophysiological Assessment

Clinical characteristics were investigated through commonly used questionnaires, which explore clinically relevant aspects for patients with chronic pain and which were validated for a Dutch-speaking population. Questionnaires include the Short Form 36 (SF-36) for general health [57], the International Physical Activity Questionnaire (IPAQ) for physical activity [58], CFS Symptom List (CSL) for ME/CFS/FM-related symptoms [59], the Widespread Pain Index (WPI) and Central Sensitization Index (CSI) for pain characteristics [60,61], and the Pan Catastrophizing Scale (PCS) and Beck Anxiety Index (BAI) for the psychological aspects of pain [62,63].

Neurophysiological measurements included pain sensitivity and descending pain modulation assessments (referred to as QST). Pain sensitivity was assessed by measuring pain thresholds to thermal and mechanical stimuli. Our protocol was designed based on previously described methods that have reliably measured hyperalgesia in chronic pain patients [64]. Pressure (kg/cm^2^) was used as the mechanical stimulus and was applied with an analogue pressure algometer (Force Dial models FDK 10 Push Pull Force Gate, Wagner Instruments, Greenwich, CT, USA) on the first inter-digital web space of the non-dominant hand. Thermal pain thresholds (°C) to cold and heat stimuli were measured in a random order at the hand (same as mechanical), the superior part of the trapezius muscle (neck), and the tibialis anterior (leg), using the TSA-II Neurosensory Analyzer (Medoc Ltd., Ramat Yishai, Israel). Three consecutive stimuli were delivered to each respective body part, and the stimulation was stopped as soon as it was perceived as painful. Thresholds were then calculated as the mean of the last two stimuli [65]. Participants were also instructed to rate the pain intensity of each threshold on a Numeric Rating Scale (NRS), ranging from 0 “No pain” to 10 “Worst pain imaginable.”

Descending pain modulation was quantified through an experimental CPM model [66]. This procedure has been validated in healthy participants, as well as patients with FM [67]. Moderately painful stimulation (rated score between 4 and 7 on a 10-point NRS) by dipping the preferred hand in a temperature-controlled (45 to 46 °C) hot-water bath (The Polystat Isotemp 4100C, Fisher Scientific, Brussels, Belgium) represented the condition stimulus and assumedly activated the descending inhibitory pain pathways. This pathway activation is accompanied by an increase in pain thresholds on the non-dominant hand in healthy people. While the dominant hand was submerged, mechanical pain sensitivity was assessed as described above. Descending modulation is thus quantified as the pain threshold difference (in °C) before and during the conditioning stimulus.

### 4.5. Blood Sample Processing and Epigenetic Analyses

A total volume of 15 mL blood was collected at each timepoint, processed (centrifuged at 3000 rpm and 4 °C for 10 min), and stored at −80 °C within 1 h of sampling until further use. Whole blood DNA was extracted using the QIAamp DNA Blood Mini Kit (Qiagen, Hilde, Germany), and the concentration was measured using a NanoDrop Spectrophotometer (Thermo Fisher Scientific, Asse, Belgium). Unmethylated cytosines were converted to uracils by bisulfite conversion using the EZ DNA Methylation-Gold kit (Zymo Research, Uden, The Netherlands), following the manufacturer’s instructions, optimized for 200 ng input DNA. Sequences of interest were then amplified by polymerase chain reaction (PCR) and subsequently sequenced, using the Q24 Pyrosequencer device (Qiagen, Hilde, Germany) and associated software. The software calculates the average methylation at each specific CpG site and provides the percentage of methylation. DNA methylation was measured on the promotor region of the *OPRM1* gene; on promoter I, exon III, promoter IV, and the coding region of exon IX of the *BDNF* gene [28]; and the promoters for the soluble and membrane-bound *COMT* variants, *S-* and *MB-COMT*, respectively, and exon IV [27]. All primers were validated by gel electrophoresis using MultiNA (Shimadzu Benelux, ‘s-Hertogenbosch, The Netherlands). Control bisulfite-converted DNA (Qiagen, Hilde, Germany) was used to perform the gel electrophoresis and for the validation of each pyrosequencing analysis. Information concerning PCR and sequencing primers and details regarding validation practices, gel electrophoresis, primer melting temperatures, and sample randomization can be found in Supplementary Tables S2 and S3 and Figures S1 and S2 [25,55,68,69,70,71].

Global DNA methylation and DNA hydromethylation were assessed using liquid chromatography and tandem mass spectrometry (LC/MS-MS), as previously described [72]. In short, genomic DNA samples (1 µg) were enzymatically hydrolysed to single deoxyribonucleotides. Digestion buffer (50 µL) containing Benzonase Nuclease, alkaline phosphatase, phosphodiesterase I, and Tris HCL solution was added to each sample and incubated at 37 °C for at least 8 h. Afterwards, 900 µL of HPLC-grade water was added to each sample. Calibration standards were made by dissolving solid reference standards of 5-methyl-2′-deoxynytidine (5mdC), 5-hydroxymethyl-2′-deoxynytidine (5hmdC), and 2′-deoxynytidine (dC) in HPLC-grade water. Global DNA methylation (5mdC) and DNA hydromethylation (5hmdC) were quantified using ultra-pressure liquid chromatography (UPLC, Waters Acquity UPLC, Waters, MA, USA) coupled to tandem mass spectrometry (MS-MS, Waters Micromass Quattro Premier Mass Spectrometer, Waters, MA, USA), with positive electrospray ionization (ESI) mode and a multiple reaction monitoring method, using argon collision gas. Per sample, 15 µL was loaded onto an Acquity UPLC BEH C_18_ Column (50 mm × 2.1 mm, 1.7 µm) that was maintained at 40 °C. For the chromatographic separation, the mobile phase consisted of a mixture of formic acid (0.1%) in water (A) and formic acid (0.1%) in acetonitrile (B) solutions. With a flow rate of 0.35 mL/min, the gradient programme was initiated. The first phase started with a linear increase from 10% to 100% B in 2 min, with a hold at 100% B for 0.1 min and subsequently returning to baseline in 0.9 min. All DNA samples were hydrolysed in triplicate and LC/MS-MS analysis in duplicate. Through interpolation with the standard curves, the absolute concentration of (hydroxy-)methylation can be calculated. Percentages were then calculated:

[%5mdC = 5mdC/(5mdc + 5hmdC + dC)] and [%5hmdC = 5hmdC/(5mdC + 5hmdC + dC)].

### 4.6. Statistical Analysis

Between-group differences in age, BMI, physical activity, and symptom outcomes were analysed using the Mann–Whitney U test (Shapiro–Wilk *p* < 0.05). Group differences were analysed using the within-subject mean of both assessments, for measures where this was relevant. In an exploratory correlation analysis, all questionnaire outcomes showed significant strong positive correlations with one another, suggesting that there is a considerable level of multicollinearity. The correlations remain stable between timepoints (see Supplementary Tables S4 and S5). We performed a regression model with Variance Inflation Factor (VIF) analysis to confirm the level of multicollinearity between our covariates. VIF analysis indicated moderate-to-severe multicollinearity between the questionnaires, with most VIF values between 3 and 5 (see Supplementary Table S6). We performed a principal component analysis (PCA) to address the multicollinearity issue, while retaining the variance of the separate outcomes. PCA resulted in the extraction of one composite pain factor consisting of all questionnaires. Kaiser–Meyer–Olkin Measure of Sampling Adequacy (KMO value) was >0.8 and Bartlett’s Test of Sphericity (Bartlett’s test) was significant, indicating a good composite component (see Supplementary Table S7 and Figure S3). Repeated-measure linear mixed models (RM-LMMs) were used to answer our main research questions. RM-LMMs account for within-subject variability while also retaining the strengths of a repeated-measures study design. To start, a basic model was fitted to assess between-group methylation differences. The basic model included “time” as the repeated measure, “group” as the fixed factor effect, “subject” as a random factor, age and BMI as covariate fixed effects, and a diagonal covariate structure. Models were corrected using the least significant difference method. Later, more advanced models were fitted to investigate the effect of other variables of interest, including symptom scores and QST measures, on *OPRM1* methylation. For each model, the goodness of fit (−2 Restricted Log Likelihood) was assessed and models with the best fit were used. Bivariate correlation analysis was applied to investigate the correlations between analysed epigenetic loci. Extended correlation tables can be found in Supplementary Tables S8 and S9. All data processing and statistical analysis were performed using IBM SPSS 29 software (Chicago, IL, USA).

## Figures and Tables

**Table 1:** Clinical characteristics of the participants. BAI, Beck Anxiety Index; BMI, body mass index; ME/CFS, myalgic encephalomyelitis/chronic fatigue syndrome; CSI, Central Sensitization Inventory; FM, fibromyalgia; IPAQ, International Physical Activity Questionnaire; METs, metabolic equivalent-minutes/week (unit of measurement for IPAQ); NA, not applicable; PCS, Pain Catastrophizing Scale; SF-36, Short Form 36, WPI, Widespread Pain Index. Values are mean ± SD. *p*-Values calculated by independent-sample Mann–Whitney U Test. * Significant *p*-values.

	Patients with ME/CFS/FM (n = 28)	Healthy Controls (n = 26)	*p*-Value
Age, years	47.95 ± 9.44	51.96 ± 10.90	0.023 *
BMI, kg/m^2^	24.34 ± 3.27	24.61 ± 4.73	0.788
Time since diagnosis, years	9.15 ± 6.51	NA	—
CFS symptom list score	112.59 ± 38.13	25.04 ± 20.15	<0.001 *
Physical activity			
IPAQ, METs	2325 ± 2255	2423 ± 2793	0.716
Pain-related factors			
SF-36 pain	59.57 ± 23.30	14.65 ± 14.58	<0.001 *
CSI	61.39 ± 12.21	24.00 ± 7.47	<0.001 *
WPI	6.50 ± 3.92	1.38 ± 1.68	<0.001 *
Psychological factors			
BAI	41.86 ± 8.47	26.54 ± 4.72	<0.001 *
PCS	21.27 ± 11.98	11.04 ± 9.45	<0.001 *

**Table 2:** Mean differences in global 5mC and 5hmC methylation, and DNA methylation at three different CpG sites and the mean methylation over phospho-Guanine dinucleotide; DNA, deoxyribonucleic acid; FM, fibromyalgia; *OPRM1*, opioid receptor mu 1. Values are reported as mean estimates ± standard error. *p*-Values calculated by repeated-measure mixed linear model with mean *OPRM1* as dependent variable, time as repeated measure, subject as random factor, group as fixed factor effect, age and BMI as covariate fixed effects, and a diagonal covariate structure. * Significant *p*-values.

DNA Methylation (%)	Patients with CFS/FM (n = 28)	Healthy Controls (n = 26)	Mean Difference (95% C.I.)	F-Test	*p*-Value
Global 5mC	3.781 ± 0.053	3.721 ± 0.053	0.061 (−0.089–0.211)	0.645	0.424
Global 5hmC	0.106 ± 0.012	0.133 ± 0.012	0.026 (−0.008–0.061)	2.385	0.127
*OPRM1* CpG1	4.191 ± 0.167	3.880 ± 0.175	0.311 (0.795–−0.172)	1.629	0.205
*OPRM1* CpG2	6.286 ± 0.259	5.458 ± 0.272	0.829 (0.076–1.581)	4.774	0.031 *
*OPRM1* CpG3	4.132 ± 0.141	3.701 ± 0.147	0.432 (0.023–0.840)	4.406	0.038 *
*OPRM1* mean	4.874 ± 0.158	4.349 ± 0.166	0.524 (0.065–0.983)	5.136	0.026 *

**Table 3:** Mean differences of objective quantitative sensory testing (QST) measures for pain. CFS, chronic fatigue syndrome; CPT, cold pain threshold; C-CPM, the effect of cold on conditioned pain modulation; HPT, heat pain threshold; H-CPM, the effect of heat on CPM; PPT, pressure pain threshold. Values are reported as mean estimates ± standard error and mean difference with 95% confidence intervals. *p*-Values calculated by repeated measure mixed linear model with the QST measure as dependent variable, time as repeated measure, subject as random factor, group as fixed factor effect, age and BMI as covariate fixed effects, and a diagonal covariate structure. * Significant *p*-values.

Quantitative Sensory Testing	Patients with CFS/FM (n = 28)	Healthy Controls (n = 26)	Mean Difference (95% C.I.)	F-Test	*p*-Value
CPT (°C)	8.314 ± 0.678	4.595 ± 0.704	3.719 (1.759–5.678)	14.178	<0.001 *
HPT (°C)	45.575 ± 0.402	47.585 ± 0.417	−2.011 (−3.172–−0.849)	11.794	<0.001 *
PPT (kg/cm^2^)	4.397 ± 0.170	5.552 ± 0.117	−1.155 (−1.647–−0.663)	21.691	<0.001 *
C-CPM (°C)	2.765 ± 0.284	1.695 ± 0.295	1.070 (0.247–1.893)	6.667	0.011 *
H-CPM (°C)	1.238 ± 0.231	0.652 ± 0.240	0.586 (−0.083–1.254)	3.022	0.085

**Table 4:** Mean between group differences of *OPRM1* methylation and the associations with the objective pain threshold measurements and conditioned pain modulation. CFS, chronic fatigue syndrome; CPT, cold pain threshold; C-CPM, the effect of cold on conditioned pain modulation; FM, fibromyalgia; HPT, heat pain threshold; H-CPM, the effect of heat on CPM; *OPRM1*, µ-opioid receptor gene; PPT, pressure pain threshold. Values are reported as mean estimates ± standard error and mean difference with 95% confidence intervals. Fixed effect values for each covariate are reported as mean estimate ± standard error. *p*-Values calculated by repeated measure mixed linear model with the mean OPRM1 methylation as dependent variable, time as repeated measure, subject as random factor, group as fixed factor effect, age and BMI as covariate fixed effects, and a diagonal covariate structure. * Significant *p*-values.

	Mean *OPRM1* Methylation					Fixed Effects		
Covariates	Patients with CFS/FM (n = 28)	Healthy Controls (n = 26)	Mean Difference (95% C.I.)	F-Test	*p*-Value	Estimate (95% C.I.)	Std. Error	*p*-Value
Quantitative sensory testing	4.866 ± 0.167	4.358 ± 0.174	0.508 (0.003–1.014)	3.990	0.049 *			
CPT						0.049 (−0.013–0.112)	0.032	0.122
HPT						0.035 (−0.074–0.143)	0.055	0.529
PPT						0.090 (−0.130–0.309)	0.111	0.420
Conditioned pain modulation	4.842 ± 0.160	4.382 ± 0.168	0.460 (−0.011–0.930)	3.763	0.055			
C-CPM						0.077 (−0.032–0.187)	0.055	0.165
H-CPM						−0.035 (−0.175–0.104)	0.070	0.616

**Table 5:** Correlation analysis of all epigenetic loci. *BDNF*, brain-derived neurotrophic factor; *MB-COMT*, membrane-bound catecholamine-O-methyltransferase; *S-COMT*, soluble catecholamine-O-transferase; *OPRM1*, µ-opioid receptor gene. Values are reported as Pearson’s r correlation coefficient. * Significant *p*-values.

		*OPRM1* Promoter I			
		Timepoint 1		Timepoint 2	
		Pearson’s r	*p*-Value	Pearson’s r	*p*-Value
*S-COMT*	Amp 1	−0.197	0.154	−0.080	0.568
	Amp 2	−0.484	<0.001 *	−0.096	0.496
	Amp 3	−0.288	0.035 *	0.025	0.856
*MB-COMT*	Amp 1	−0.188	0.416	0.063	0.654
	Amp 2	−0.07	0.608	0.037	0.791
	Amp 3	−0.033	0.814	0.204	0.143
COMT Exon IV		−0.285	0.037 *	−0.180	0.197
*BDNF* Promoter I	Amp 1	0.680	<0.001 *	0.561	<0.001 *
	Amp 2	0.680	<0.001 *	0.606	<0.001 *
	Amp 3	0.376	0.005 *	0.746	<0.001 *
	Amp 4	0.037	0.788	0.096	0.511
*BDNF* Exon III	Amp 1	0.592	<0.001 *	0.357	0.009 *
	Amp 2	0.628	<0.001 *	0.613	<0.001 *
*BDNF* Promoter IV	Amp 1	0.212	0.123	0.237	0.088
	Amp 2	0.180	0.193	0.022	0.875
*BDNF* Exon IX	Amp 1	−0.061	0.661	−0.261	0.061
	Amp 2	−0.258	0.059	−0.028	0.844

## Data Availability

Due to privacy restrictions, the data presented in this study are available on request from the corresponding author.

## Supplementary Materials

The following supporting information can be downloaded at: https://www.mdpi.com/article/10.3390/ijms27020826/s1.

## Abbreviations

The following abbreviations are used in this manuscript:

5hmC	5-Hydroxymethylcytosine
5hmdC	5-hydroxymethyl-2′-deoxynytidine
5hmdC	Global DNA hydroxy methylation
5mC	5-Methylcytosine
5mdC	Global DNA methylation
ARC	American College of Rheumatology Criteria
BAI	Beck Anxiety Index
BDNF	Brain-derived neurotrophic factor
BMI	Body mass index
C-CPM	Cold effect on conditioned pain modulation
COMT	Catecholamine-O-methyltransferase
CpG	Cytosine-phosphate-Guanine dinucleotide
CPM	Conditioned pain modulation
CPT	Cold pain thresholds
CSI	Central sensitization inventory
CSL	CFS symptom list for ME/CFS-related symptoms
DNA	Deoxyribonucleic acid
DNMT3a	DNA methyltransferase 3a
EM-Seq	Enzymatic methyl-sequencing
FM	Fibromyalgia
fMRI	Functional magnetic resonance imaging
H-CPM	Heat effect on conditioned pain modulation
HDAC1	Histone deacetylase 1
HPLC	High-pressure liquid chromatography
HPT	Heat pain thresholds
IPAQ	International physical activity questionnaire
LC/MS-MS	Liquid chromatography and tandem mass spectrometry
MB-COMT	Membrane-bound COMT
MBD1	Methyl-CpG-binding domain 1
ME/CFS	Myalgic encephalomyelitis/chronic fatigue syndrome
MeCP2	Methyl-CpG-binding proteins
METs	Metabolic equivalents
MOR	µ-Opioid receptor 1
mRNA	Messenger RNA
NRS	Numeric Rating Scale
OPRM1	µ-Opioid receptor 1 gene
PCA	Principal component analysis
PCR	Polymerase chain reaction
PCS	Pain catastrophizing scale
PET	Positron emission tomography
PPT	Pressure pain threshold
QST	Quantitative sensory testing
RM-LMM	Repeated-measures linear mixed model
S-COMT	Soluble COMT
SF-36	Short-form 36 questionnaire
TAPSß	TET-assisted pyridine borane sequencing
VIF	Variance inflation factor
WPI	Widespread Pain Index
