# Supplementary material for: Hypermethylation of OPRM1: Deregulation of the Endogenous Opioid Pathway in Myalgic Encephalomyelitis/Chronic Fatigue Syndrome and Fibromyalgia
Source: Int J Mol Sci. 2026 Jan 14;27(2):826. doi: 10.3390/ijms27020826 (PMC12840744; doi:10.3390/ijms27020826)
Supplement: Supplementary file 1 [file ijms-27-00826-s001.zip › ijms-4052861-supplementary.pdf]

Supplementary Materials

Hypermethylation of OPRM1: Deregulation of the Endogenous Opioid Pathway in Myalgic Encephalomyelitis/Chronic Fatigue Syndrome and Fibromyalgia

Screening outcomes of the medication plan notes

Patients were instructed to not start new therapies 6 weeks before the first test moment and to not use nicotine, alcohol and caffeine 24h before the test moment. Additionally, all participants were screened for comorbidities and current medication plans were noted.

Drug Class	Healthy Controls (N)	Patients (N)
Analgesics (non-opioid)	2	7
Opioids	0	5
Antidepressants	0	12
Anticonvulsants / neuropathic pain	0	2
Benzodiazepines / sedative-hypnotics	0	7
Cardiovascular medications	5	2
Endocrine / hormonal therapy	4	4
Antidiabetic medication	0	2
Gastrointestinal medications	0	7
Immunomodulatory / anti-inflammatory	1	3
Antibiotics / antimicrobials	1	2
Vitamins & supplements	7	11

Table S1. The table represents the most prominent medication classes, and which participants were taking them during the assessment.

PCR and sequencing primer design and validation

For COMT DNA methylation analyses, we used PyroMark Assay Design Software 2.0 to design forward, reverse, and sequencing primers to assess DNA methylation in three regions of interest – MB-COMT, S-COMT, and Exon IV (where two important polymorphisms are located – rs4818 and rs4680). Regions of interest were selected based on previous literature, which showed these regions to influence gene expression [1, 2]. On the contrary, OPRM1 primers for the region we were interested in (Exon I, as previously published) were commercially available and validated by QIAGEN (Heidelberg, Germany)[3, 4]. For the BDNF gene, we first searched the literature to find previously designed primers using similar methodology (PCR-pyrosequencing) for our regions of interest – promoters I and IV, and exon III and IX [5-7]. Primers targeting the desired regions of Promoter I and Exon III were purchased from Qiagen. Primers for Promoter IV and Exon IX were designed using the PyroMark Assay Design Software 2.0. For an overview of the primer sequences, target sequences, annealing temperatures, and a visual representation of the location of the amplified sequences, refer to Table S2 and Figure S1. Using a gradient PCR Device (Veriti 96-Well Thermal Cycler, Applied Biosystems, ThermoFisher Scientific, Belgium), we undertook the validation process to detect which annealing temperature worked best.

PCR cycling protocol was set as follows:

- One activation step: 95°C for 15 minutes,
- 45 cycles including 3 steps
    - 30 seconds at 94°C for denaturation,
    - 30 seconds at the annealing temperature detected during the validation procedure (See Table S1)
    - 30 seconds extension step at 72°C
- One final extension step of 10 minutes at 72°C
- Final hold at 4°C for ∞

**MB-COMTa**	GTTGAGGGGA TTAGGAGGG	CCCCAATTTCCC CACCTA	CCCATCCTACCTA CT	CTG**CG**CCC**CGCG**C**CGCG**CCC**CG**CACC C**CG**CC**CG**CCA**CG**GCCTG**CG**TC**CG**CCA C**CG**GAAG**CG**	13	58
**MB-COMTb**	GGGTAGTTTTAGT TGTTTTAGAAGTTT	ACCCCATCCTAC CTACTAC	CACCCAAAACCCC CTCCTAATC	C**CG**CAG**CG**CCAC**CG**CCATTGC**CG**CCA T**CG**T**CG**TG	6	58
**MB-COMTc**	TGGGGTAGTTA GGGTTGT	ATCTAACCAACC TCTCACTCTC	CAACCCTCTCACC TCTCC	C**CG**C**CGCG**CTGCCTG**CG**C**CG**GAC**CG** GGG**CG**GGTCCAGTCC**CG**GG**CG**GGC**C** **G**T**CGCG**G	11	58
**S-COMTa**	GGATGGGTTGT AGGATGAAT	ACATTTCTAAAC CTTACCCCTCTA	AGTAATATAGTTG TTAATAGTAGA	GGG**CG**ATGGTGGCACTCCAAGCAAA GGGG**CG**TGTGGGTGCTGCAGGAGG AGCACAGAGCACTGG**CG**CCCCTCCCC TCC**CG**CCCTGCAGATGCC**CG**G	4	54
**S-COMTb**	GTGGATGGGTT GTAGGATGA	AAACCCCCCCTCTA CTATTAACAACTAT	GGGTTGTAGGATG AAT	CTGTGCCTTAT**CG**GCTGGAA**CG**AGT	2	54
**S-COMTc**	TGTTTATGGGTGA TATTAAGGAGTAG	TCATAACCCACT CCTTCTACT	AAATATCAATAACC TCCAAC	**CG**CATCCTGAACCA**CG**TGCTGCAGCA TG**CG**GAGCC**CG**GGAA**CG**CACAGAG**C** **G**TGCTGGAGGCCATTGACACCTACTG **CG**	6	54
**Exon IV**	TGGGGGTTTATT GTGGTTAT	AACTATAAAACCC TCACTAAACTACTA	ACACACCTTATCCT TC	CAGCTGTG**CG**CATGGCC**CG**CCTGCTG TCACCAGGGG**CG**AGGCTCATCACCAT **CG**AGATCAACCC**CG**ACTGTGC**CG**CCA TCACCCAG**CG**GATGGTGGATTT**CG**CT GG**CG**TGAAGGACAAGGTGTGCATGC CTGACC**CG**TTGTCAGACCTGGAAAAA GGGC**CG**GCTGTGGGCAGGGAGGGC	6	56

				ATG**CG**CACTTTGTCCTCCCCACCAGG TGTTCACACCA**CG**TTCACT		
**OPRM1**	PM00024794	PM00024794	PM00024794	TTGCTGG**CG**TT**YG**TGGGGRCAG**CG**C	3	56
**BDNF Prom Ia**	P6_PM00155526	P6_PM00155526	P6_PM00155526	**CG**CAGTCATAACTTCATTCAACTCAGC **CG**CT**CG**A	3	56
**BDNF Prom Ib**	P8_PM00155540	P8_PM00155540	P8_PM00155540	CTGCATG**CG**T**CG**AAG**CGCG**A	4	58
**BDNF Prom Ic**	P9_PM00155547	P9_PM00155547	P9_PM00155547	TTACTTTC**CG**CCAACA**CG**TGACCTCTT **CG**CTTCCCAGCTTG**CG**T	5	58
**BDNF Exon IIIa**	P4_ PM00155512	P4_ PM00155512	P4_ PM00155512	C**CG**GCC**CG**GCTGGGGAY**CG**GAG**CGC G**GTCT**CG**GC	6	56
**BDNF Exon IIIb**	P5_ PM00155519	P5_ PM00155519	P5_ PM00155519	G**CG**CC**CG**AAACT**CGCG**GGGA	4	58
**BDNF Prom IVa**	GGGTTGGAAGTGA AAATATTTGTAAA	CCCCATCAACCAA A AACTCCATTTAATC	TTTGGTAATTCG TGTATTAGAGT	GTCTATTT**CG**AGGCAG**CG**GAGGTATC ATATGACAG**CG**CA**CG**TCAAGGCAC**CG** TGGAGCCCTCT**CG**T	6	56
**BDNF Prom IVb**	GGGTTGGAAGTGA AAATATTTGTAAA	CCCCATCAACCAA A AACTCCATTTAATC	GTGGATTTTTAT TTATTTTTTTAT	TCAC**CGCG**GAGAGGGCTGCTCT**CG**C TGC**CG**CTCCCCC**CG**G**CG**AACTAGCAT GAAATCTCCCTGCCTCTGC**CG**A	7	56
**BDNF Exon IXa**	ATGAAGGTTGTTTT TATGAAAGAAGTA	ACCCACTCA CTAATACTA	GTTGTAAATAT GTTTATGAGGG	**CG**CTCAGCTCCCCT**CG**G**CG**GGCAGG GTCAGAGTGG**CG**C**CG**	5	56
**BDNF Exon IXb**	ATGAAGGTTGTTTT TATGAAAGAAGTA	ACCCACTCA CTAATACTA	AGGTTTAAG AGGTTTGA	**CG**TGTACAAGTCTG**CG**TCCTTATTGTT TTCTTCATTGGGC**CG**AACTTTCTGGTC CTCATCCAACAGCTCTTCTATCA**YG**TG TT**CG**	5	56

Table S2. Primers for DNA methylation analyses of the COMT, OPRM1, and BDNF genes. Using the PyroMark Assay Design Software 2.0, primer pairs were designed for the MB- and S- COMT promoters and Exon IV, and for BDNF promoter IX and Exon IX (coding region). For OPRM1, BDNF Promoter I and Exon III Primer pairs were purchased from QIAGEN. Primer sequences were not specified, we report in the table the product catalog number and the sequence to analyse. Temperatures (°C) refer to the annealing temperatures used for PCR amplification.

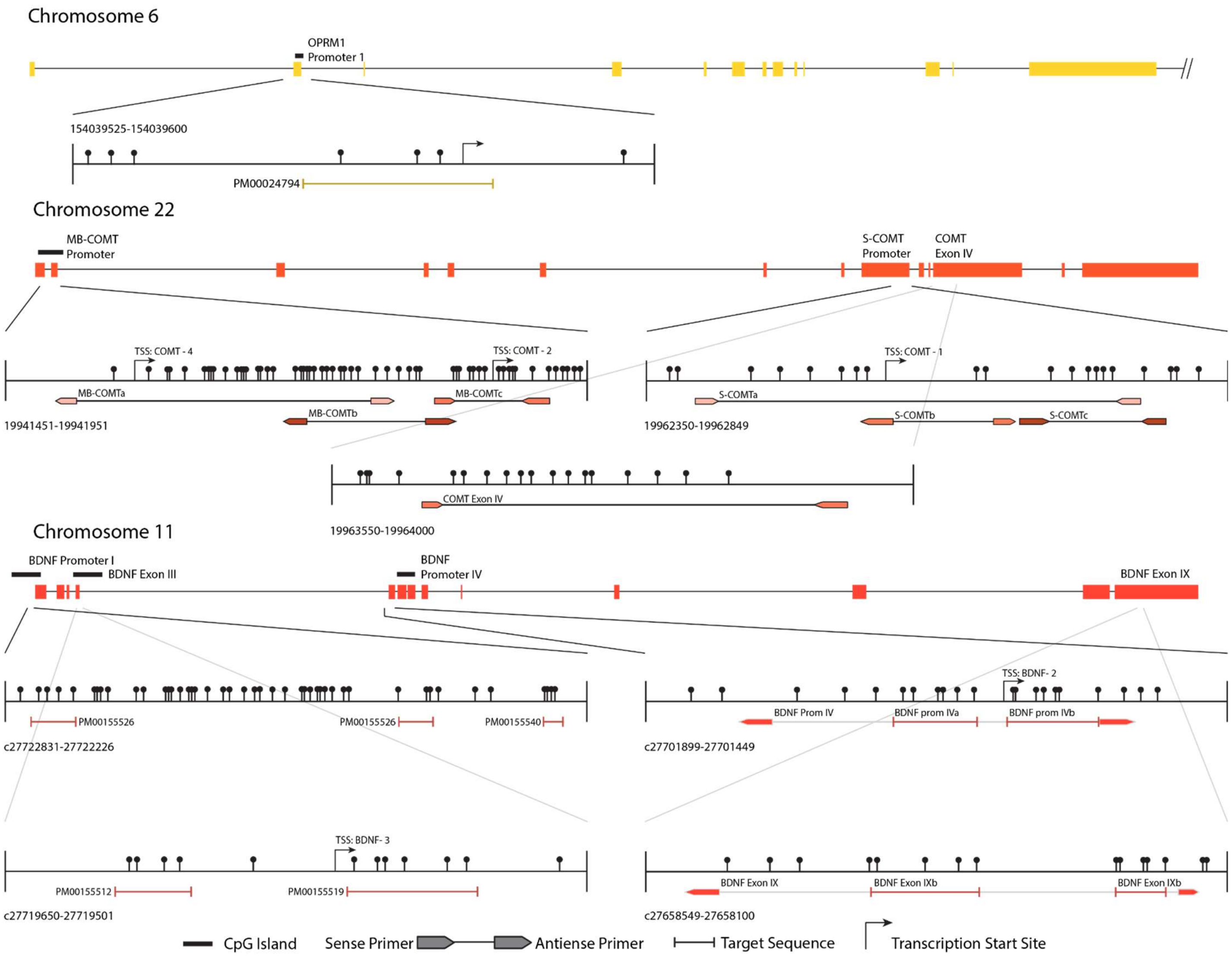

Figure S1. Gives an overview of the genetic structure, and a representation of the amplicon location and primer binding sites.

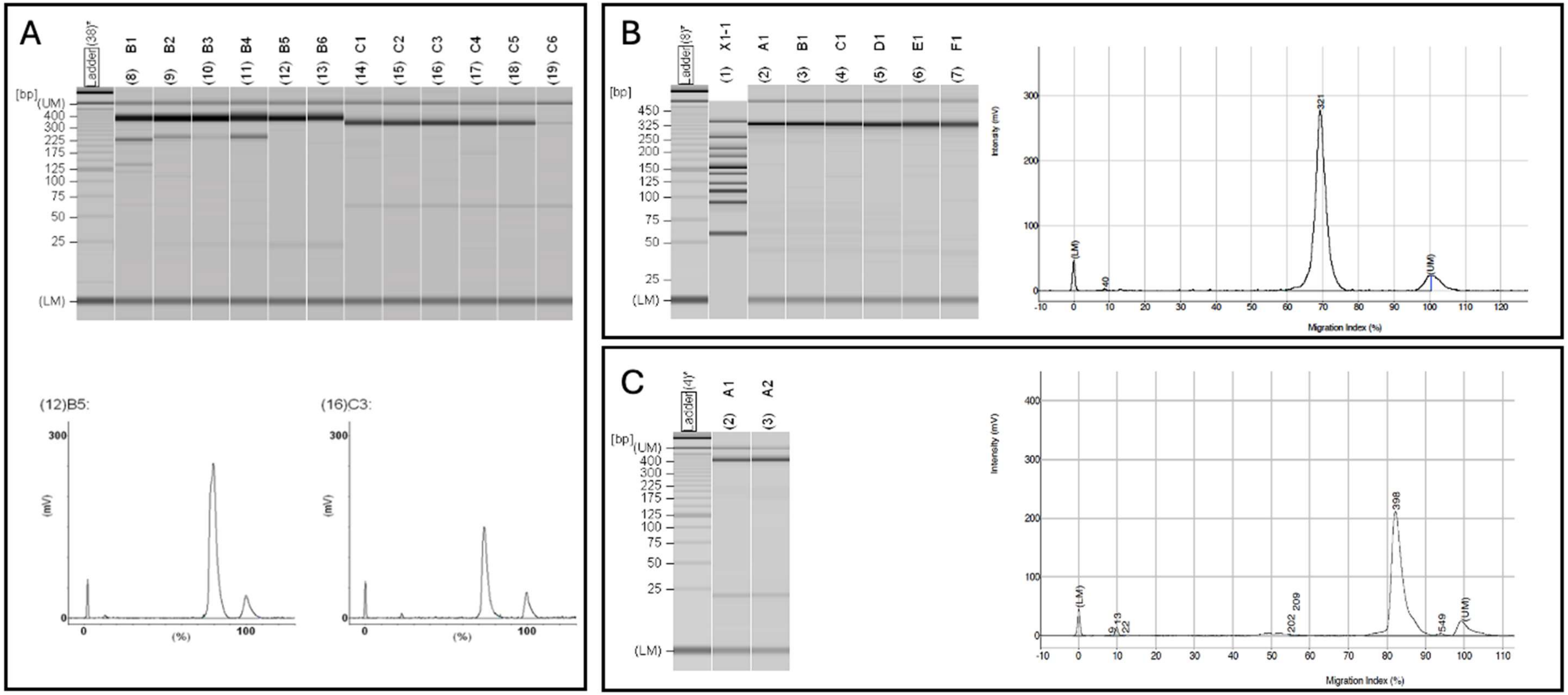

Figure S2. Primer validation procedure using a gradient PCR Device. Per target gene, only two examples are shown to clarify the procedure. All primers underwent the same validation procedure. Box A represents MB-COMT (B-columns) and S-COMT (C-columns), Box B BDNF promoter IV and Box C BDNF Exon IX. Each gel electrophoresis image is accompanied by an electropherogram for the selected temperatures. The first column of each gel represents a molecular weight ladder. Both for COMT and BDNF promoter IV, numbers 1-6 refer to different temperatures: 50°C, 52°C, 54°C, 56°C, 58°C, 60°C. For BDNF Exon IX, only two columns showed positive signals where the software was able to give valid outputs, A1 and A2 refer to 54°C and 56°C, respectively.

Randomisation and positive controls for pyrosequencing analyses

Pyrosequencing was performed on 24-well plates using a Q24 Pyrosequencer device (Qiagen, Hilde, Germany). Sample randomisation was performed in order to reduce the bias that might have accumulated during the lab procedures, and to ensure that each plate would include both patients and controls in random order. DNA positive (highly methylated) controls used to validate the pyrosequencing. We included two control wells per analysis, in different plates. One of the two controls in the MB-COMTa plate did not work and variability could not be calculated. Positive controls were expected to be highly methylated (≥80%) and with low variability (up to 5%).

**MB-COMTa**	71.85%	/	/
**MB-COMTb**	86.49%	86.99%	0.50%
**MB-COMTc**	86.18%	82.33%	3.85%
**S-COMTa**	94.00%	90.18%	3.82%
**S-COMTb**	100.00%	98.61%	1.39%
**S-COMTc**	84.52%	84.77%	0.22%
**Exon IV**	72.56%	74.37%	1.81%
**OPRM1**	93.28%	94.57%	1.29%
**BDNF Prom Ia**	84.86%	83.63%	1.23%
**BDNF Prom Ib**	76.41%	78.29%	1.88%
**BDNF Prom Ic**	88.66%	87.81%	0.85%
**BDNF Exon IIIa**	92.10%	93.26%	1.16%
**BDNF Exon IIIb**	92.50%	94.10%	1.60%
**BDNF Prom IVa**	95.64%	95.10%	0.44%
**BDNF Prom IVb**	91.97%	90.54%	1.43%
**BDNF Exon IXa**	96.71%	96.87%	0.16%
**BDNF Exon IXb**	81.75%	81.10%	0.65%

Table S3. Mean methylation for each positive control in the two wells, and the inter-sample variability. Only MB-COMTa and Exon IV, and BDNF prom Ib, did not reach 80% methylation. However, variation was small so this will unlikely impact on the analyses. Plus, even in the case of higher variation, all samples are randomised, and the repeated measure design allowed us to test each subject twice.

Correlation analysis multicollinearity questionnaires

In an exploratory correlation analysis, all questionnaire outcomes showed significant strong positive correlations with one another, suggesting that there is a considerable level of multicollinearity. The correlations remain stable between timepoints.

Time = 1

Correlations[a]

			MET (IPQA)	CSL total	Central Sensitisation (CSI)	Widespread Pain Index	SF-36 Pain Subscale	PCS total	Pain Vigilance (PVAQ)	BAI total
Spearman's rho	MET (IPQA)	Correlation Coefficient	1.000	.154	.141	.091	.087	-.098	-.003	.064
		Sig. (2-tailed)	.	.267	.308	.515	.530	.481	.981	.646
		N	54	54	54	54	54	54	54	54
	CSL total	Correlation Coefficient	.154	1.000	.913**	.745**	.745**	.481**	.416**	.812**
		Sig. (2-tailed)	.267	.	<.001	<.001	<.001	<.001	.002	<.001
		N	54	54	54	54	54	54	54	54
	Central Sensitisation (CSI)	Correlation Coefficient	.141	.913**	1.000	.770**	.765**	.553**	.464**	.822**
		Sig. (2-tailed)	.308	<.001	.	<.001	<.001	<.001	<.001	<.001
		N	54	54	54	54	54	54	54	54
	Widespread Pain Index	Correlation Coefficient	.091	.745**	.770**	1.000	.754**	.548**	.333*	.645**
		Sig. (2-tailed)	.515	<.001	<.001	.	<.001	<.001	.014	<.001
		N	54	54	54	54	54	54	54	54
	SF-36 Pain Subscale	Correlation Coefficient	.087	.745**	.765**	.754**	1.000	.678**	.459**	.747**
		Sig. (2-tailed)	.530	<.001	<.001	<.001	.	<.001	<.001	<.001
		N	54	54	54	54	54	54	54	54
	PCS total	Correlation Coefficient	-.098	.481**	.553**	.548**	.678**	1.000	.634**	.611**
		Sig. (2-tailed)	.481	<.001	<.001	<.001	<.001	.	<.001	<.001
		N	54	54	54	54	54	54	54	54
	Pain Vigilance (PVAQ)	Correlation Coefficient	-.003	.416**	.464**	.333*	.459**	.634**	1.000	.512**
		Sig. (2-tailed)	.981	.002	<.001	.014	<.001	<.001	.	<.001
		N	54	54	54	54	54	54	54	54
	BAI total	Correlation Coefficient	.064	.812**	.822**	.645**	.747**	.611**	.512**	1.000
		Sig. (2-tailed)	.646	<.001	<.001	<.001	<.001	<.001	<.001	.
		N	54	54	54	54	54	54	54	54

**. Correlation is significant at the 0.01 level (2-tailed).
*. Correlation is significant at the 0.05 level (2-tailed).
a. Time = 1

Table S4. Correlation analysis for all symptom related questionnaire outcomes at timepoint 1.

Time = 2

Correlations[a]

			MET (IPQA)	CSL total	Central Sensitisation (CSI)	Widespread Pain Index	SF-36 Pain Subscale	PCS total	Pain Vigilance (PVAQ)	BAI total
Spearman's rho	MET (IPQA)	Correlation Coefficient	1.000	.079	.118	.096	.215	.182	.173	.178
		Sig. (2-tailed)	.	.569	.394	.490	.119	.188	.212	.199
		N	54	54	54	54	54	54	54	54
	CSL total	Correlation Coefficient	.079	1.000	.834**	.699**	.751**	.473**	.381**	.803**
		Sig. (2-tailed)	.569	.	<.001	<.001	<.001	<.001	.004	<.001
		N	54	54	54	54	54	54	54	54
	Central Sensitisation (CSI)	Correlation Coefficient	.118	.834**	1.000	.770**	.765**	.553**	.464**	.831**
		Sig. (2-tailed)	.394	<.001	.	<.001	<.001	<.001	<.001	<.001
		N	54	54	54	54	54	54	54	54
	Widespread Pain Index	Correlation Coefficient	.096	.699**	.770**	1.000	.754**	.548**	.333*	.700**
		Sig. (2-tailed)	.490	<.001	<.001	.	<.001	<.001	.014	<.001
		N	54	54	54	54	54	54	54	54
	SF-36 Pain Subscale	Correlation Coefficient	.215	.751**	.765**	.754**	1.000	.678**	.459**	.721**
		Sig. (2-tailed)	.119	<.001	<.001	<.001	.	<.001	<.001	<.001
		N	54	54	54	54	54	54	54	54
	PCS total	Correlation Coefficient	.182	.473**	.553**	.548**	.678**	1.000	.634**	.577**
		Sig. (2-tailed)	.188	<.001	<.001	<.001	<.001	.	<.001	<.001
		N	54	54	54	54	54	54	54	54
	Pain Vigilance (PVAQ)	Correlation Coefficient	.173	.381**	.464**	.333*	.459**	.634**	1.000	.401**
		Sig. (2-tailed)	.212	.004	<.001	.014	<.001	<.001	.	.003
		N	54	54	54	54	54	54	54	54
	BAI total	Correlation Coefficient	.178	.803**	.831**	.700**	.721**	.577**	.401**	1.000
		Sig. (2-tailed)	.199	<.001	<.001	<.001	<.001	<.001	.003	.
		N	54	54	54	54	54	54	54	54

**. Correlation is significant at the 0.01 level (2-tailed).
*. Correlation is significant at the 0.05 level (2-tailed).
a. Time = 2

Table S5. Correlation analysis for all symptom related questionnaire outcomes at timepoint 2.

Principal component analysis

We performed a regression model with Variance Inflation Factor (VIF) analysis to confirm the level of multicollinearity between our covariates. VIF analysis indicated moderate-to-severe multicollinearity between the questionnaires, with most VIF values between 3 and 5.

Coefficients[a]

Model		Unstandardized Coefficients		Standardized Coefficients			Collinearity Statistics	
		B	Std. Error	Beta	t	Sig.	Tolerance	VIF
1	(Constant)	4.352	.548		7.942	<.001		
	CSL total	.007	.005	.295	1.436	.154	.208	4.811
	Central Sensitisation (CSI)	-.019	.013	-.342	-1.503	.136	.169	5.900
	Widespread Pain Index	.001	.044	.004	.027	.978	.429	2.333
	SF-36 Pain Subscale	.005	.007	.129	.720	.473	.273	3.669
	PCS total	-.036	.016	-.355	-2.214	.029	.341	2.935
	Pain Vigilance (PVAQ)	-.004	.011	-.050	-.397	.692	.560	1.787
	BAI total	.036	.022	.308	1.612	.110	.240	4.161

a. Dependent Variable: OPRM1_mean

Table S6. Regression model with VIF analysis to detect multicollinearity between the questionnaires.

We performed a principal component analysis (PCA) to address the multicollinearity issue, while retaining the variance of the separate outcomes. PCA resulted in the extraction of one composite pain factor consisting of all questionnaires. Kaiser-Meyer-Olkin Measure of Sampling Adequacy (KMO value) was >0.8 and the Bertlett's Test of Sphericity (Bertlett's test) was significant, indicating a good composite component.

KMO and Bartlett's Test

Kaiser-Meyer-Olkin Measure of Sampling Adequacy.		.900
Bartlett's Test of Sphericity	Approx. Chi-Square	856.187
	df	45
	Sig.	<.001

Communalities

	Initial	Extraction
Central Sensitisation (CSI)	1.000	.840
Widespread Pain Index	1.000	.596
SF-36 Pain Subscale	1.000	.753
PCS total	1.000	.415
BAI total	1.000	.782
CSL_pain	1.000	.775
CSL_fatigue	1.000	.670
CSL_sleep	1.000	.421
CSL_cognitive	1.000	.721
CSL_immune	1.000	.488

Extraction Method: Principal Component Analysis.

Total Variance Explained

Component	Initial Eigenvalues			Extraction Sums of Squared Loadings		
	Total	% of Variance	Cumulative %	Total	% of Variance	Cumulative %
1	6.462	64.617	64.617	6.462	64.617	64.617
2	.895	8.948	73.565			
3	.663	6.634	80.199			
4	.462	4.616	84.815			
5	.415	4.153	88.968			
6	.358	3.583	92.551			
7	.282	2.822	95.373			
8	.216	2.160	97.533			
9	.135	1.350	98.883			
10	.112	1.117	100.000			

Extraction Method: Principal Component Analysis.

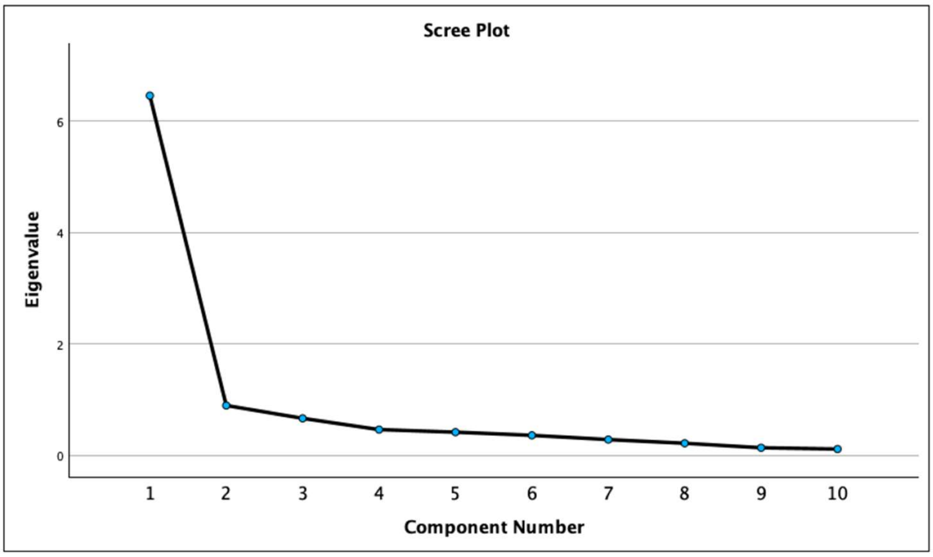

Component Matrix[a]

	Component 1
Central Sensitisation (CSI)	.917
Widespread Pain Index	.772
SF-36 Pain Subscale	.868
PCS total	.644
BAI total	.884
CSL_pain	.880
CSL_fatigue	.819
CSL_sleep	.649
CSL_cognitive	.849
CSL_immune	.699

Extraction Method: Principal Component Analysis.

a. 1 components extracted.

Table S7 and Figure S3. All tables describe the process of the PCA. The Figure S2, is a Scree Plot, which gives visual confirmation of the successful extraction of a single component.

Extended correlation analyses tables: OPRM1-COMT-BDNF

Correlations[a]																			
		OPRM1_mean	S_COMT_Mean1	S_COMT_Mean2	S_COMT_Mean3	MB_COMT_Mean1	MB_COMT_Mean2	MB_COMT_Mean3	Mean	Promotor 1_Amp1	Promoter 1_Amp2	Promoter 1_Amp3	Promoter 1_Amp4	Intron 3_Amp1	Intron 3_Amp2	Exon 9_Amp1	Exon 9_Amp2	Promoter 4_Amp1	Promoter 4_Amp2
OPRM1_mean	Pearson Correlation	1	-0.197	-.484**	-.288*	-0.118	-0.071	-0.033	-.285*	.680**	.608**	.376**	0.037	.592**	.628**	-0.061	-0.258	0.212	0.180
	Sig. (2-tailed)		0.154	0.000	0.035	0.416	0.608	0.814	0.037	0.000	0.000	0.005	0.788	0.000	0.000	0.661	0.059	0.123	0.193
	N	54	54	54	54	50	54	54	54	54	53	54	54	54	54	54	54	54	54
S_COMT_Mean1	Pearson Correlation	-0.197	1	.617**	-0.125	-0.080	-0.078	0.033	-0.109	0.035	-0.148	-0.012	-0.036	-.440**	-.316*	-0.210	0.151	0.098	0.049
	Sig. (2-tailed)	0.154		0.000	0.369	0.582	0.575	0.813	0.432	0.802	0.291	0.934	0.797	0.001	0.020	0.127	0.276	0.480	0.726
	N	54	54	54	54	50	54	54	54	54	53	54	54	54	54	54	54	54	54
S_COMT_Mean2	Pearson Correlation	-.484**	.617**	1	-0.067	-0.022	-0.052	0.090	0.068	-0.234	-.288*	-0.011	-0.024	-.561**	-.380**	-.323*	.300*	-0.168	-0.163
	Sig. (2-tailed)	0.000	0.000		0.629	0.882	0.709	0.518	0.628	0.089	0.036	0.937	0.862	0.000	0.005	0.017	0.027	0.225	0.240
	N	54	54	54	54	50	54	54	54	54	53	54	54	54	54	54	54	54	54
S_COMT_Mean3	Pearson Correlation	-.288*	-0.125	-0.067	1	0.138	0.092	0.043	0.227	-.413**	-.325*	-.394**	-0.105	-0.169	-0.119	.314*	0.075	0.129	-0.210
	Sig. (2-tailed)	0.035	0.369	0.629		0.341	0.507	0.756	0.099	0.002	0.018	0.003	0.448	0.221	0.390	0.021	0.588	0.353	0.127
	N	54	54	54	54	50	54	54	54	54	53	54	54	54	54	54	54	54	54
MB_COMT_Mean1	Pearson Correlation	-0.118	-0.080	-0.022	0.138	1	0.010	.533**	0.175	-0.054	-0.025	-0.076	0.021	-0.094	0.160	-0.143	-0.002	0.115	0.118
	Sig. (2-tailed)	0.416	0.582	0.882	0.341		0.947	0.000	0.223	0.709	0.863	0.600	0.884	0.515	0.268	0.320	0.988	0.426	0.415
	N	50	50	50	50	50	50	50	50	50	49	50	50	50	50	50	50	50	50
MB_COMT_Mean2	Pearson Correlation	-0.071	-0.078	-0.052	0.092	0.010	1	-0.097	0.070	-0.132	0.109	-.463**	-0.015	-0.090	-0.138	0.175	0.172	-0.065	0.020
	Sig. (2-tailed)	0.608	0.575	0.709	0.507	0.947		0.486	0.614	0.340	0.437	0.000	0.912	0.517	0.320	0.205	0.213	0.640	0.885
	N	54	54	54	54	50	54	54	54	54	53	54	54	54	54	54	54	54	54
MB_COMT_Mean3	Pearson Correlation	-0.033	0.033	0.090	0.043	.533**	-0.097	1	-0.176	0.007	-0.106	0.010	0.030	0.147	-0.046	-.274*	-0.266	-0.051	0.045
	Sig. (2-tailed)	0.814	0.813	0.518	0.756	0.000	0.486		0.204	0.961	0.448	0.944	0.830	0.288	0.742	0.045	0.052	0.714	0.746
	N	54	54	54	54	50	54	54	54	54	53	54	54	54	54	54	54	54	54
Mean	Pearson Correlation	-.285*	-0.109	0.068	0.227	0.175	0.070	-0.176	1	-.358**	-0.146	-0.062	0.081	-.339*	-.276*	-.293*	0.164	-.416**	0.133
	Sig. (2-tailed)	0.037	0.432	0.628	0.099	0.223	0.614	0.204		0.008	0.296	0.659	0.561	0.012	0.043	0.031	0.237	0.002	0.339
	N	54	54	54	54	50	54	54	54	54	53	54	54	54	54	54	54	54	54
Promotor 1_Amp1	Pearson Correlation	.680**	0.035	-0.234	-.413**	-0.054	-0.132	0.007	-.358**	1	.665**	.347*	-0.032	.414**	.485**	-0.019	-0.062	.289*	0.146
	Sig. (2-tailed)	0.000	0.802	0.089	0.002	0.709	0.340	0.961	0.008		0.000	0.010	0.820	0.002	0.000	0.891	0.655	0.034	0.293
	N	54	54	54	54	50	54	54	54	54	53	54	54	54	54	54	54	54	54
Promoter 1_Amp2	Pearson Correlation	.608**	-0.148	-.288*	-.325*	-0.025	0.109	-0.106	-0.146	.665**	1	0.256	-0.017	.296*	.468**	-0.043	-0.061	0.219	0.266
	Sig. (2-tailed)	0.000	0.291	0.036	0.018	0.863	0.437	0.448	0.296	0.000		0.064	0.903	0.032	0.000	0.759	0.663	0.116	0.055
	N	53	53	53	53	49	53	53	53	53	53	53	53	53	53	53	53	53	53
Promoter 1_Amp3	Pearson Correlation	.376**	-0.012	-0.011	-.394**	-0.076	-.463**	0.010	-0.062	.347*	0.256	1	-0.001	0.086	0.156	-.478**	0.011	0.124	0.072
	Sig. (2-tailed)	0.005	0.934	0.937	0.003	0.600	0.000	0.944	0.659	0.010	0.064		0.996	0.536	0.259	0.000	0.938	0.374	0.605
	N	54	54	54	54	50	54	54	54	54	53	54	54	54	54	54	54	54	54
Promoter 1_Amp4	Pearson Correlation	0.037	-0.036	-0.024	-0.105	0.021	-0.015	0.030	0.081	-0.032	-0.017	-0.001	1	0.125	0.194	-0.087	0.067	-0.156	0.091
	Sig. (2-tailed)	0.788	0.797	0.862	0.448	0.884	0.912	0.830	0.561	0.820	0.903	0.996		0.370	0.159	0.531	0.632	0.261	0.514
	N	54	54	54	54	50	54	54	54	54	53	54	54	54	54	54	54	54	54
Intron 3_Amp1	Pearson Correlation	.592**	-.440**	-.561**	-0.169	-0.094	-0.090	0.147	-.339*	.414**	.296*	0.086	0.125	1	.539**	0.229	-.324*	0.215	-0.078
	Sig. (2-tailed)	0.000	0.001	0.000	0.221	0.515	0.517	0.288	0.012	0.002	0.032	0.536	0.370		0.000	0.096	0.017	0.119	0.575
	N	54	54	54	54	50	54	54	54	54	53	54	54	54	54	54	54	54	54
Intron 3_Amp2	Pearson Correlation	.628**	-.316*	-.380**	-0.119	0.160	-0.138	-0.046	-.276*	.485**	.468**	0.156	0.194	.539**	1	0.070	-0.168	0.266	0.171
	Sig. (2-tailed)	0.000	0.020	0.005	0.390	0.268	0.320	0.742	0.043	0.000	0.000	0.259	0.159	0.000		0.613	0.226	0.052	0.216
	N	54	54	54	54	50	54	54	54	54	53	54	54	54	54	54	54	54	54
Exon 9_Amp1	Pearson Correlation	-0.061	-0.210	-.323*	.314*	-0.143	0.175	-.274*	-.293*	-0.019	-0.043	-.478**	-0.087	0.229	0.070	1	-0.032	0.263	-.278*
	Sig. (2-tailed)	0.661	0.127	0.017	0.021	0.320	0.205	0.045	0.031	0.891	0.759	0.000	0.531	0.096	0.613		0.821	0.055	0.042
	N	54	54	54	54	50	54	54	54	54	53	54	54	54	54	54	54	54	54
Exon 9_Amp2	Pearson Correlation	-0.258	0.151	.300*	0.075	-0.002	0.172	-0.266	0.164	-0.062	-0.061	0.011	0.067	-.324*	-0.168	-0.032	1	0.142	-0.197
	Sig. (2-tailed)	0.059	0.276	0.027	0.588	0.988	0.213	0.052	0.237	0.655	0.663	0.938	0.632	0.017	0.226	0.821		0.307	0.153
	N	54	54	54	54	50	54	54	54	54	53	54	54	54	54	54	54	54	54
Promoter 4_Amp1	Pearson Correlation	0.212	0.098	-0.168	0.129	0.115	-0.065	-0.051	-.416**	.289*	0.219	0.124	-0.156	0.215	0.266	0.263	0.142	1	-0.208
	Sig. (2-tailed)	0.123	0.480	0.225	0.353	0.426	0.640	0.714	0.002	0.034	0.116	0.374	0.261	0.119	0.052	0.055	0.307		0.132
	N	54	54	54	54	50	54	54	54	54	53	54	54	54	54	54	54	54	54
Promoter 4_Amp2	Pearson Correlation	0.180	0.049	-0.163	-0.210	0.118	0.020	0.045	0.133	0.146	0.266	0.072	0.091	-0.078	0.171	-.278*	-0.197	-0.208	1
	Sig. (2-tailed)	0.193	0.726	0.240	0.127	0.415	0.885	0.746	0.339	0.293	0.055	0.605	0.514	0.575	0.216	0.042	0.153	0.132	
	N	54	54	54	54	50	54	54	54	54	53	54	54	54	54	54	54	54	54

**. Correlation is significant at the 0.01 level (2-tailed).

*. Correlation is significant at the 0.05 level (2-tailed).

a. Time = 1

Table S8. Extended Correlation table to investigate the correlations between analysed epigenetic loci at timepoint 1.

Correlations[a]

		OPRM1_mean	S_COMT_Mean1	S_COMT_Mean2	S_COMT_Mean3	MB_COMT_Mean1	MB_COMT_Mean2	MB_COMT_Mean3	Mean	Promotor 1_Amp1	Promoter 1_Amp2	Promoter 1_Amp3	Promoter 1_Amp4	Intron 3_Amp1	Intron 3_Amp2	Exon 9_Amp1	Exon 9_Amp2	Promoter 4_Amp1	Promoter 4_Amp2
OPRM1_mean	Pearson Correlation	1	-0.080	-0.096	0.025	0.063	0.037	0.204	-0.180	.561**	.606**	.746**	0.096	.357**	.613**	-0.261	-0.028	0.237	0.022
	Sig. (2-tailed)		0.568	0.496	0.856	0.654	0.791	0.143	0.197	0.000	0.000	0.000	0.511	0.009	0.000	0.061	0.844	0.088	0.875
	N	53	53	53	53	53	53	53	53	53	53	51	49	53	53	52	53	53	53
S_COMT_Mean1	Pearson Correlation	-0.080	1	0.251	0.010	-0.093	-0.064	0.038	0.069	-0.045	-.356**	-0.081	0.021	-.396**	-0.092	0.073	0.035	0.115	0.025
	Sig. (2-tailed)	0.568		0.067	0.943	0.501	0.644	0.783	0.618	0.744	0.008	0.574	0.884	0.003	0.508	0.608	0.804	0.408	0.857
	N	53	54	54	54	54	54	54	54	54	54	51	50	54	54	52	53	54	54
S_COMT_Mean2	Pearson Correlation	-0.096	0.251	1	-0.187	.334*	0.239	.298*	-0.239	0.056	-0.163	-0.274	0.109	-.314*	-0.097	0.222	-0.083	-0.145	0.154
	Sig. (2-tailed)	0.496	0.067		0.175	0.014	0.082	0.029	0.082	0.687	0.240	0.052	0.451	0.021	0.487	0.113	0.554	0.295	0.265
	N	53	54	54	54	54	54	54	54	54	54	51	50	54	54	52	53	54	54
S_COMT_Mean3	Pearson Correlation	0.025	0.010	-0.187	1	-0.087	-0.139	-0.181	-0.084	-0.229	-.309*	-0.036	0.062	-0.013	-0.238	0.158	-0.119	0.078	-0.182
	Sig. (2-tailed)	0.856	0.943	0.175		0.529	0.317	0.191	0.546	0.095	0.023	0.802	0.671	0.923	0.083	0.262	0.398	0.573	0.188
	N	53	54	54	54	54	54	54	54	54	54	51	50	54	54	52	53	54	54
MB_COMT_Mean1	Pearson Correlation	0.063	-0.093	.334*	-0.087	1	.646**	.856**	-0.260	0.148	-0.121	0.052	0.220	0.030	0.049	-0.084	0.084	-0.036	0.257
	Sig. (2-tailed)	0.654	0.501	0.014	0.529		0.000	0.000	0.058	0.285	0.383	0.719	0.124	0.829	0.726	0.556	0.549	0.798	0.060
	N	53	54	54	54	54	54	54	54	54	54	51	50	54	54	52	53	54	54
MB_COMT_Mean2	Pearson Correlation	0.037	-0.064	0.239	-0.139	.646**	1	.676**	-0.202	0.170	-0.073	0.012	0.205	0.083	0.141	-0.037	0.251	0.201	0.140
	Sig. (2-tailed)	0.791	0.644	0.082	0.317	0.000		0.000	0.143	0.220	0.598	0.935	0.153	0.551	0.309	0.797	0.069	0.145	0.311
	N	53	54	54	54	54	54	54	54	54	54	51	50	54	54	52	53	54	54
MB_COMT_Mean3	Pearson Correlation	0.204	0.038	.298*	-0.181	.856**	.676**	1	-0.234	.289*	-0.084	0.165	.284*	0.108	0.137	-0.040	0.110	0.152	0.253
	Sig. (2-tailed)	0.143	0.783	0.029	0.191	0.000	0.000		0.088	0.034	0.545	0.247	0.045	0.439	0.322	0.781	0.433	0.274	0.065
	N	53	54	54	54	54	54	54	54	54	54	51	50	54	54	52	53	54	54
Mean	Pearson Correlation	-0.180	0.069	-0.239	-0.084	-0.260	-0.202	-0.234	1	-0.184	0.004	-0.137	-0.245	0.249	-0.057	.318*	-0.256	0.090	-0.151
	Sig. (2-tailed)	0.197	0.618	0.082	0.546	0.058	0.143	0.088		0.184	0.978	0.336	0.086	0.069	0.682	0.022	0.064	0.520	0.277
	N	53	54	54	54	54	54	54	54	54	54	51	50	54	54	52	53	54	54
Promotor 1_Amp1	Pearson Correlation	.561**	-0.045	0.056	-0.229	0.148	0.170	.289*	-0.184	1	.403**	.679**	0.264	0.166	.504**	-0.246	0.013	0.190	.335*
	Sig. (2-tailed)	0.000	0.744	0.687	0.095	0.285	0.220	0.034	0.184		0.002	0.000	0.064	0.229	0.000	0.078	0.927	0.168	0.013
	N	53	54	54	54	54	54	54	54	54	54	51	50	54	54	52	53	54	54
Promoter 1_Amp2	Pearson Correlation	.606**	-.356**	-0.163	-.309*	-0.121	-0.073	-0.084	0.004	.403**	1	.443**	0.099	.345*	.456**	-.397**	0.018	0.020	0.003
	Sig. (2-tailed)	0.000	0.008	0.240	0.023	0.383	0.598	0.545	0.978	0.002		0.001	0.494	0.011	0.001	0.004	0.897	0.883	0.984
	N	53	54	54	54	54	54	54	54	54	54	51	50	54	54	52	53	54	54
Promoter 1_Amp3	Pearson Correlation	.746**	-0.081	-0.274	-0.036	0.052	0.012	0.165	-0.137	.679**	.443**	1	0.017	.376**	.580**	-.376**	-0.045	0.128	-0.066
	Sig. (2-tailed)	0.000	0.574	0.052	0.802	0.719	0.935	0.247	0.336	0.000	0.001		0.908	0.007	0.000	0.007	0.755	0.372	0.647
	N	51	51	51	51	51	51	51	51	51	51	51	47	51	51	50	51	51	51
Promoter 1_Amp4	Pearson Correlation	0.096	0.021	0.109	0.062	0.220	0.205	.284*	-0.245	0.264	0.099	0.017	1	0.073	0.012	0.049	-0.016	.479**	.446**
	Sig. (2-tailed)	0.511	0.884	0.451	0.671	0.124	0.153	0.045	0.086	0.064	0.494	0.908		0.616	0.932	0.741	0.911	0.000	0.001
	N	49	50	50	50	50	50	50	50	50	50	47	50	50	50	48	49	50	50
Intron 3_Amp1	Pearson Correlation	.357**	-.396**	-.314*	-0.013	0.030	0.083	0.108	0.249	0.166	.345*	.376**	0.073	1	.297*	-0.088	-0.030	0.242	-0.041
	Sig. (2-tailed)	0.009	0.003	0.021	0.923	0.829	0.551	0.439	0.069	0.229	0.011	0.007	0.616		0.029	0.535	0.830	0.078	0.767
	N	53	54	54	54	54	54	54	54	54	54	51	50	54	54	52	53	54	54
Intron 3_Amp2	Pearson Correlation	.613**	-0.092	-0.097	-0.238	0.049	0.141	0.137	-0.057	.504**	.456**	.580**	0.012	.297*	1	-0.267	0.173	.303*	0.087
	Sig. (2-tailed)	0.000	0.508	0.487	0.083	0.726	0.309	0.322	0.682	0.000	0.001	0.000	0.932	0.029		0.056	0.216	0.026	0.530
	N	53	54	54	54	54	54	54	54	54	54	51	50	54	54	52	53	54	54
Exon 9_Amp1	Pearson Correlation	-0.261	0.073	0.222	0.158	-0.084	-0.037	-0.040	.318*	-0.246	-.397**	-.376**	0.049	-0.088	-0.267	1	-0.265	0.158	0.183
	Sig. (2-tailed)	0.061	0.608	0.113	0.262	0.556	0.797	0.781	0.022	0.078	0.004	0.007	0.741	0.535	0.056		0.058	0.263	0.194
	N	52	52	52	52	52	52	52	52	52	52	50	48	52	52	52	52	52	52
Exon 9_Amp2	Pearson Correlation	-0.028	0.035	-0.083	-0.119	0.084	0.251	0.110	-0.256	0.013	0.018	-0.045	-0.016	-0.030	0.173	-0.265	1	0.106	-0.037
	Sig. (2-tailed)	0.844	0.804	0.554	0.398	0.549	0.069	0.433	0.064	0.927	0.897	0.755	0.911	0.830	0.216	0.058		0.449	0.792
	N	53	53	53	53	53	53	53	53	53	53	51	49	53	53	52	53	53	53
Promoter 4_Amp1	Pearson Correlation	0.237	0.115	-0.145	0.078	-0.036	0.201	0.152	0.090	0.190	0.020	0.128	.479**	0.242	.303*	0.158	0.106	1	.333*
	Sig. (2-tailed)	0.088	0.408	0.295	0.573	0.798	0.145	0.274	0.520	0.168	0.883	0.372	0.000	0.078	0.026	0.263	0.449		0.014
	N	53	54	54	54	54	54	54	54	54	54	51	50	54	54	52	53	54	54
Promoter 4_Amp2	Pearson Correlation	0.022	0.025	0.154	-0.182	0.257	0.140	0.253	-0.151	.335*	0.003	-0.066	.446**	-0.041	0.087	0.183	-0.037	.333*	1
	Sig. (2-tailed)	0.875	0.857	0.265	0.188	0.060	0.311	0.065	0.277	0.013	0.984	0.647	0.001	0.767	0.530	0.194	0.792	0.014	
	N	53	54	54	54	54	54	54	54	54	54	51	50	54	54	52	53	54	54

**. Correlation is significant at the 0.01 level (2-tailed).

*. Correlation is significant at the 0.05 level (2-tailed).

a. Time = 2

Table S9. Extended Correlation table to investigate the correlations between analysed epigenetic loci at timepoint 2.

References

1. Polli, A., et al., *DNA Methylation and Brain-Derived Neurotrophic Factor Expression Account for Symptoms and Widespread Hyperalgesia in Patients With Chronic Fatigue Syndrome and Comorbid Fibromyalgia.* Arthritis & Rheumatology, 2020/11/01. **72**(11).
2. Swift-Scanlan, T., et al., *Comprehensive interrogation of CpG island methylation in the gene encoding COMT, a key estrogen and catecholamine regulator.* BMC Medical Genomics, 2014. **7**(1): p. 5.
3. Chidambaran, V., et al., *DNA methylation at the mu-1 opioid receptor gene (OPRM1) promoter predicts preoperative, acute, and chronic postsurgical pain after spine fusion.* Pharmacogenomics and Personalized Medicine, 2017. **Volume 10**: p. 157-168.
4. Oertel, B.G., et al., *Genetic-epigenetic interaction modulates mu-opioid receptor regulation.* Hum Mol Genet, 2012. **21**(21): p. 4751-60.
5. Keller, S., et al., *Increased BDNF Promoter Methylation in the Wernicke Area of Suicide Subjects.* Archives of General Psychiatry, 2010. **67**(3): p. 258.
6. Stenz, L., et al., *BDNF promoter I methylation correlates between post-mortem human peripheral and brain tissues.* Neuroscience Research, 2015. **91**: p. 1-7.
7. Xu, X., et al., *A significant association between BDNF promoter methylation and the risk of drug addiction.* Gene, 2016. **584**(1): p. 54-59.
